# PKC isoforms in hematopoietic lineages and myeloid/lymphoid leukemias: mechanistic insights and therapeutic prospects

**DOI:** 10.3389/fonc.2026.1891770

**Published:** 2026-08-06

**Authors:** Xu Wang, Chunming Wang, Suling Li, Kaibei Zhang, Anqi Liu, Xuyan Deng, Qingju Bian, Shijia Lu, Jinwen Sima

**Affiliations:** 1 North Henan Medical University, Xinxiang, China; 2 Zhejiang Chinese Medical University, Hangzhou, China; 3 Jilin University, Changchun, China; 4 Zhengzhou Central Hospital, Zhengzhou, China

**Keywords:** drug resistance, hematopoiesis, isoform specificity, leukemia, precision therapy, protein kinase C

## Abstract

Leukemia remains a major clinical challenge due to high rates of drug resistance and recurrence, necessitating the identification of novel therapeutic targets. Protein kinase C (PKC) family, a central signaling hub regulating cell proliferation, differentiation, and apoptosis, has been implicated in hematological malignancies for decades. However, the context-dependent and often contradictory functions of PKC isoforms across different hematopoietic lineages have hindered the development of effective targeted therapies. Here, we systematically review the roles of PKC isoforms in normal hematopoiesis and the pathogenesis of myeloid and lymphoid leukemias, adopting a dual lineage- and isoform-centric perspective to resolve the long-standing functional paradox of PKC signaling. We first delineate the lineage-specific regulatory functions of distinct PKC subfamilies in hematopoietic stem cell maintenance and lineage commitment. We then comprehensively analyze the dual oncogenic and tumor-suppressive roles of individual PKC isoforms in major leukemia subtypes, highlighting their critical involvement in drug resistance and leukemia stem cell survival. Finally, we evaluate current PKC-targeted therapeutic strategies and propose future directions for lineage-specific precision therapy. This review provides a unified framework for understanding PKC signaling in hematopoiesis and leukemia, offering novel insights into overcoming therapeutic resistance and improving patient outcomes.

## 1 Introduction

Protein Kinase C (PKC) is a phospholipid-dependent serine/threonine protein kinase (1, 2). PKC is composed of a single polypeptide chain, and 12 distinct PKC isoforms have been identified in mammalian cells (3). As a multifunctional protein, PKC plays a key role in various intracellular signaling pathways involved in development, differentiation, proliferation, apoptosis, and carcinogenesis (4). Due to their high sequence homology, PKC isoforms share many structural and functional similarities. However, subtle differences in amino acid sequences within the full-length molecule, especially in the N-terminal region, lead to notable variations among PKC isoforms in terms of domain configuration, cofactor requirements, and sensitivity to activators (5). The functional divergence among these isoforms provides a rationale for classifying them into three subfamilies: the classical subfamily (cPKC: α, βI, βII, and γ), whose members contain two functional lipid-sensing domains (C1 and C2) and require calcium, diacylglycerol (DAG) or phorbol esters, as well as phosphatidylserine (PS) for activation; the novel subfamily (nPKC: δ, ϵ, η, and θ), whose members possess a non-functional C2 domain and do not require calcium for activation; and the atypical subfamily (aPKC: ι and ζ), whose members lack part of the C1 domain entirely and also lack the C2 domain, rendering them insensitive to DAG, phorbol esters, and calcium (3).

The major pathogenic signaling pathways in leukemia are well characterized. In AML, FLT3-ITD mutations cause constitutive receptor tyrosine kinase activation, driving downstream STAT5, PI3K/AKT, and RAS/RAF/MEK cascades, leading to uncontrolled proliferation and poor prognosis, making FLT3 a key therapeutic target (6, 7). The JAK-STAT pathway is frequently hyperactivated; phosphorylated STAT3/5 dimerize and translocate to the nucleus, upregulating Bcl-2, c-MYC, and cyclin D, while also recruiting epigenetic modifiers (e.g., EZH2, DNA methyltransferases) to sustain leukemic stem cells and synergize with FLT3 mutations (8). The PI3K/AKT/mTOR axis regulates proliferation, metabolism, survival, and cell death (9) its overactivation, found in >50% of acute leukemias (especially myeloid), promotes disease progression and drug resistance. In the bone marrow microenvironment, stromal-derived CXCL12 and VEGF bind CXCR4/VEGFR on leukemic cells, activating PI3K/AKT and conferring chemoresistance (10); additionally, immune checkpoint molecules like PD-L1 suppress T cell function, facilitating immune escape. The MAPK pathway, commonly activated by NRAS/KRAS mutations, leads to ERK-mediated phosphorylation of transcription factors (e.g., ELK1) and drives cell cycle/survival genes; it also crosstalks with FLT3 and KIT signaling, representing a potential therapeutic avenue (6).

PKC isoforms also play critical roles in the aforementioned signaling pathways (FLT3, JAK-STAT, PI3K/AKT, and MAPK) that drive leukemogenesis. In the NF-κB pathway, PKCθ is recruited to the immune synapse upon TCR activation, phosphorylates CARD11, facilitates CBM complex assembly, and activates IKK kinase (11). PKCι/λ interacts with Par6 via the PB1 domain, activating the Rac1/cdc42–IKKβ axis in lung cancer. In MAPK/ERK signaling, PKCϵ activates H-Ras through RasGRP3, triggering Raf/MEK/ERK (12) Similarly, PKCα promotes pancreatic cancer proliferation via Raf-1/ERK (13) while PKCθ modulates the immunosuppressive microenvironment in T-cell lymphoma through NF-κB/AP-1 (14, 15) and also inhibits Treg suppressive function (16). Based on these mechanisms, several targeted agents are under development. The broad-spectrum inhibitor midostaurin, which targets FLT3 (as noted above), has prolonged overall survival in FLT3-mutant AML (17); the PKCβ-selective inhibitor enzastaurin is in Phase III trials for diffuse large B-cell lymphoma (18).

Although research on PKC in cancer has been extensive, the complex nature of PKC signaling and its context-dependent roles across different diseases makes it hard to generalize. In the specific context of leukemia, while existing literature has covered certain aspects of PKC biology, a systematic synthesis that comprehensively integrates its lineage-specific and subtype-specific functions remains relatively limited. This review aims to summarize PKC’s role in normal hematopoiesis and its involvement in hematological disorders, both benign and malignant. It seeks to provide a theoretical foundation for researchers to develop precise PKC-targeted therapies. Given the conflicting evidence of PKC subtypes having opposing effects in different leukemias, the review uses ‘lineage’ and ‘subtype’ frameworks to systematically compare and reconcile these contradictions, exploring the molecular basis and assessing the potential for specific therapeutic approaches.

## 2 The structure, regulation and involved signaling pathways of PKC

Based on the structural characteristics and regulatory mechanisms of PKC family, they can be classified into three major categories: classical PKC (cPKC), novel PKC (nPKC), and atypical PKC (aPKC). This classification was initially established by the Nishizuka team in the 1980s through biochemical and molecular cloning techniques (19).

Conventional protein kinase C (cPKC) encompasses four subtypes: α, βI, βII, and γ. A notable structural characteristic of cPKC is the presence of two conserved domains, designated as C1 and C2. The C1 domain comprises approximately 50 amino acids and includes six cysteine residues and two histidine residues, which together form a double zinc finger structure essential for diacylglycerol (DAG) binding (20). The lower part of the ligand-binding pocket of the C1 domain is hydrophilic and can form hydrogen bonds with the glycerol backbone of diacylglycerol; while the upper part is hydrophobic and suitable for accommodating the fatty acid chain of diacylglycerol (21). The C2 domain has a typical β-triangular structure and contains multiple calcium ion-binding sites, which are important for calcium-dependent activation (22). In the resting state, cPKC binds to the substrate pocket within the C4 domain via its N-terminal pseudosubstrate sequence, maintaining the enzyme in an inactive state (23). When cells are stimulated, second messengers (diacylglycerol, calcium ions) bind to the regulatory domain, causing conformational changes and releasing the catalytic domain, thereby activating the kinase (24). Specifically, the C2 domain of cPKC first binds to Ca^2+^, guiding the enzyme to bind to the phosphatidylserine-containing plasma membrane. Subsequently, the membrane-bound C1 domain captures DAG, releasing the auto-inhibition, and the catalytic domain is fully exposed and phosphorylated to achieve a stable conformation (5, 25).

nPKC is comprised of four subtypes: δ, ϵ, η, and θ. The primary structural distinction between nPKC and cPKC resides in the sequence of the C1 and C2 domains within the protein’s linear sequence. Notably, the C2 domain of nPKC is deficient in the critical calcium-binding acidic residues, rendering it incapable of activation via calcium. Nevertheless, its activation is contingent upon the direct interaction of diacylglycerol (DAG) with the C1 domain, facilitating membrane recruitment and alleviation of self-inhibition. Empirical research has demonstrated that nPKC activation is independent of calcium signaling, instead being mediated through the direct association of the C1 domain with DAG for membrane recruitment (26). Similar to cPKC, the newly synthesized nPKC requires phosphorylation at the conserved threonine residues (such as Thr505 in PKCδ) on the activation loop by the upstream kinase PDK1 to acquire full catalytic activity. Additionally, nPKC also undergoes autophosphorylation at the Turn motif and hydrophobic sites, and these phosphorylation events jointly stabilize the active conformation of the kinase (27).

aPKC consists of two subtypes, namely the ζ subtype and the ι/λ subtype. Its structural feature is that only a part of the C1 domain is retained, and the C2 domain is completely absent (22). This makes aPKC insensitive to DAG, phorbol ester, and Ca^2+^. aPKC is mainly activated through protein-protein interactions. The regulation of aPKC mainly relies on protein-protein interactions mediated by the PB1 domain. PKCζ can form a complex with p62 or Par6 through the PB1 domain and be recruited to specific cellular subregions to exert its function (28).

The differences in structure among different PKC subtypes determine their sensitivity to activating signals. cPKC can be activated only when both Ca^2+^ and DAG are present, nPKC can be activated only by DAG, while aPKC does not depend on these two second messengers. This structural feature provides a theoretical basis for designing specific subtype drugs targeting different PKCs.

## 3 The role of PKC in hematopoiesis physiology

PKC enzymes function as critical signal transducers in hematopoietic development and immune cell function. During the process of myeloid hematopoiesis, specific PKC subtypes play crucial roles as regulators of myeloid cell fate determination, differentiation programs, and effector functions, usually functioning downstream of cytokines. The expression of different PKC subtypes forms a complex regulatory network to ensure the normal development and function of granulocytes, monocytes, erythrocytes, and megakaryocytes. This has been demonstrated in studies of zebrafish embryonic development, human leukemia cell lines, and primary human cells (28–30).

### 3.1 The role of PKC in hematopoietic stem cells

Hematopoietic stem cells (HSCs) serve as the progenitors for all blood cell lineages, characterized by their capacities for self-renewal and multipotent differentiation. Long-term (LT) HSCs exhibit substantial self-renewal potential, albeit with restricted short-term differentiation, thereby maintaining a lifelong reservoir of stem cells. Conversely, short-term (ST) HSCs demonstrate a pronounced propensity for differentiation, playing a crucial role in the rapid replenishment of diverse cell types required by the hematopoietic system over a shorter temporal framework. The molecular signals controlling the maintenance of long-term HSCs and their transformation into short-term HSCs are rather complex, and PKC plays an important role (32). In the CD34^+^ long-term HSC population, the expression of specific PKC genes (namely Prkca (PKCα), Prkce (PKCϵ), Prkch (PKCη), and Prkcq (PKCθ)) is higher than that in the short-term HSC population (33). These specific PKC subtypes may potentially play a role in maintaining the state of long-term HSCs. However, the functional requirements of a single PKC do not seem to be irreplaceable, as the absence of a single subtype gene does not cause a catastrophic impact on the hematopoietic process (34). Atypical PKC (aPKC) (PKCζ and PKCλ/ι) are dispensable in the hematopoietic process whether in a steady state or under stress conditions, as their absence does not damage the self-renewal or lineage reconstruction of HSCs (35). This means that either other PKCs will compensate for their absence, or there are mechanisms that do not require PKC to maintain the function of HSCs.

### 3.2 The role of PKC in granulocyte and monocyte cells

Neutrophils develop from hematopoietic stem cells, and this process involves a complex differentiation process in which various PKC subtypes play stage-specific roles. The translocation of PKCϵ and PKCλ/ι to the cell surface to the cell surface is closely related to the acquisition of neutrophil characteristics after growth factor stimulation (32). In mature neutrophils, the activation of PKC can play an antibacterial defense function. During phagocytosis, specific PKC subtypes exhibit regional activation patterns: when neutrophils encounter IgG-coated particles as a stimulus, PKCβI and βII transfer to the phagosome, while PKCδ and PKCζ are recruited to the secretory granules, indicating that there is a complex division of labor among PKC family members to coordinate the maturation of phagosomes and granule release (36). Components of the respiratory burst response are largely dependent on PKC-mediated phosphorylation of the NADPH oxidase components for the killing of intracellular pathogens. Multiple PKC subtypes, including PKCα, PKCβII, PKCδ, and PKCζ, can phosphorylate the p47phox subunit of NADPH, thereby promoting its assembly with p22phox and subsequently generating superoxide radicals (37). Due to the overlapping functions of these different PKC functions, this explains why single PKC gene knockout models only show partial defects in reactive oxygen species (ROS) production.

The PKC signaling pathway plays an important role in the differentiation of hematopoietic progenitor cells into the monocytic lineage. The stimulation of monocyte/macrophage colony-stimulating factor (M-CSF) can induce the continuous production of diacylglycerol and promote the nuclear translocation of PKCα in granulocyte/macrophage progenitor cells. Constitutively activated PKCα with ectopic expression is sufficient to drive macrophage differentiation (38). During the development of dendritic cells, the binding of GM-CSF and IL-4 can induce rapid and sustained phosphorylation of PKCδ, thereby phosphorylating the transcription factor PU.1, enhancing its DNA-binding ability and promoting the differentiation of dendritic cells (39). Each subtype of PKC also functions as a key signaling mediator in the pattern recognition receptor pathway. Macrophages deficient in PKCϵ have weakened responses to lipopolysaccharide (TLR4 ligand), impaired NF-κB activation, reduced production of pro-inflammatory cytokines, and are more susceptible to Gram-negative bacterial infections (32). Similarly, the response mediated by TLR1/2 to bacterial lipopeptides depends on PKCα, while PKCδ plays an irreplaceable role in Dectin-1 signaling for fungal pathogens. The anti-fungal function of PKCδ is also manifested in the inhibition of intracellular bacteria, as macrophages lacking PKCδ can produce complete cytokines but cannot restrict monocyte chlamydia within phagosomes (32).

### 3.3 The role of PKC in megakaryocytes and erythrocytes

The differentiation of megakaryocytes/erythrocyte progenitor cells is dynamically regulated by PKC signaling. PKCϵ functions as a positive regulator in the early lineage determination, and it is induced by TPO to promote the differentiation of hematopoietic stem cells into megakaryocytes (30). PKCδ plays a balancing negative regulatory role, and it is related to the apoptosis and cycle arrest of various cells (30). In primary myelofibrosis (PMF), both PKCϵ is high and PKCδ is low (dysregulation of differentiation), while in primary thrombocytosis (ET), PKCϵ is low and PKCδ is high (enhanced platelet production) (30). In the process of human megakaryocyte generation, the dynamic expression of PKCδ is opposite to that of PKCϵ, and their balance is crucial for the full maturation of megakaryocytes and the generation of platelets; PKCδ and PKCϵ, as functionally coupled entities, play opposite roles in platelet production, and their balanced regulation can strongly affect platelet production, which may be achieved through the Bax and Bcl-xL pathways; *in vitro* experiments have shown that in both normal and pathological conditions, the PKCϵ/PKCδ system can be corrected to treat thrombocytopenia and thrombocytosis (30). Thrombopoietin (TPO) induces the early expression of PKCϵ in human CD34+ cells, and this expression gradually decreases in the subsequent differentiation stage. Forced maintenance of PKCϵ expression delays the maturation of megakaryocytes (31). At the molecular level, PKCϵ cooperates with GATA-1 to activate the ITGA2B promoter, which encodes the megakaryocyte-specific integrin glycoprotein IIb. Other PKC subtypes cannot replace this function, indicating that there is subtype specificity in megakaryocyte differentiation (31). In platelets, PKCα activates the Rap1 GTPase in nucleated cells to mediate platelet integrin αIIbβ3, mediating the coagulation factor signaling pathway; while PKCβ interacts with the β3 chain to regulate the cytoskeleton reorganization of platelets in response to the binding of fibrinogen induced by coagulation factors (40, 41). And PKCθ continuously binds to αIIbβ3 and promotes the signal transduction induced by fibrinogen through the interaction with Btk and Syk kinases (42). PKCα or PKCβ deficiency hinders the formation of thrombi under flow conditions, while PKCθ or PKCδ deficiency promotes aggregation abnormally, indicating that the novel PKC isoforms have opposite effects in platelet function (32). PKCα and PKCβ are necessary for the outward signal transduction through integrin αIIbβ3 via the fibrinogen-induced platelet aggregation (41, 43), while PKCθ and PKCδ have opposite effects on thrombosis formation under shear flow (44).

During the development of red blood cells, multiple stages rely on precise regulation by PKC. In the erythropoiesis-driven differentiation process of human and mouse cells by erythropoietin, the expression of PKCϵ gradually increases, which promotes the survival of red blood cells by counteracting TRAIL-mediated cell apoptosis (31). The mechanism of action of PKC in the erythropoiesis process mainly lies in post-transcriptional regulation. The PKC-mediated RNA-binding protein ELAVL1 (HuR) phosphorylates at the Ser219 and Ser316 sites, facilitating its nuclear-cytoplasmic shuttling, thereby stabilizing gata1 mRNA and promoting erythrocyte differentiation (29). This pathway is conserved across different species, which has also been confirmed in the zebrafish model. Inhibition of PKC or mutation of ELAVL1 impairs embryonic erythrocyte generation. The crucial importance of PKC signaling in erythrocyte development has also been further confirmed by pharmacological studies. PKC inhibitors H-7, H-8, and statospinosin significantly reduced the colony formation of normal late erythroid progenitor cells (CFU-e) (29).

### 3.4 The role of PKC in immune cells

In the process of lymphoid hematopoiesis, PKC is a key component of cytokine signal transduction during lymphocyte generation (31). In the early lymphocyte generation process, various PKC subtypes participate in the differentiation of hematopoietic stem cells into lymphocyte precursors. For the development of early B cells, the IL-7 receptor functions through a non-classical pathway, which involves diacylglycerol (DAG) generated by PLCγ, thereby activating PKC and independently stimulating mTOR activity in an Akt/TSC/Rheb axis-independent manner (31). This indicates that PKC enzymes can function independently of the conventional pathway;This PLCγ/PKC/mTOR signaling pathway is crucial for IL-7-induced metabolic reprogramming, cell cycle progression, and the differentiation of B cell precursors (31). The deficiency of PLCγ1 and PLCγ2 hinders the development of B cells at the pre-B cell stage, which is similar to the defects observed in IL-7R or Jak3 deficiency, highlighting the criticality of this pathway (31). Each subtype of PKC also plays specific roles in different lymphocyte subsets. PKCθ and PKCη exhibit functional redundancy during positive selection of thymocytes (45, 46), while PKCα supports pre-TCR signal transduction during the DN3 stage (47). The activation of mature T cells involves the transfer of PKCθ to the immune synapse, where it binds to CD28 and activates NF-κB through CARMA1 (48). In regulatory T cells, PKCη binds to CTLA-4 to promote inhibitory function (49). In effector T cells, PKCθ is crucial for Th2 and Th17 differentiation (50–53), while PKCδ negatively regulates mast cell degranulation (54). In NK cells, PKCθ, PKCδ, and PKCη regulate cytokine production and cytotoxicity mediated by activating receptors (55–57).

**Figure 1:**
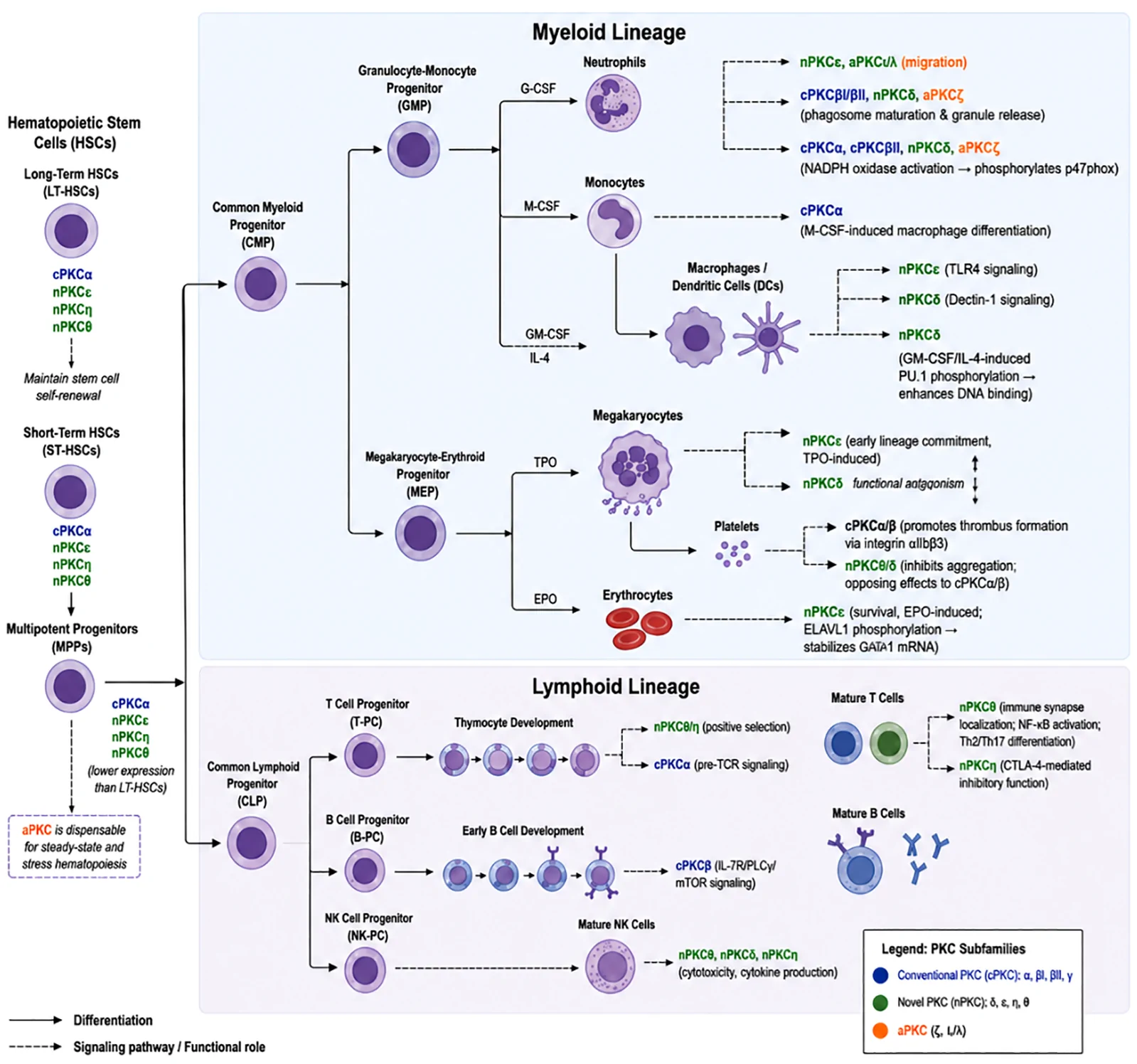
The role of PKC isoforms in normal hematopoiesis. (This schematic diagram illustrates the lineage-specific regulatory roles of protein kinase C (PKC) isoforms during physiological hematopoiesis, starting from hematopoietic stem cells (HSCs) and progressing through multipotent progenitors (MPPs) to fully differentiated myeloid and lymphoid lineage cells. Long-term HSCs (LT-HSCs) maintain self-renewal capacity through the expression of cPKCα, nPKCϵ, nPKCη, and nPKCθ, with expression levels decreasing in short-term HSCs (ST-HSCs) and MPPs. Notably, atypical PKC (aPKC) isoforms are dispensable for both steady-state and stress hematopoiesis. In the myeloid lineage, distinct PKC isoforms regulate granulocyte, monocyte/macrophage, megakaryocyte, and erythrocyte development and function, with a particularly well-characterized functional antagonism between nPKCϵ (positive regulator) and nPKCδ (negative regulator) during megakaryopoiesis. In the lymphoid lineage, PKC signaling controls early B-cell development, thymocyte selection, and the effector functions of mature T cells, natural killer (NK) cells, and B cells. Solid arrows indicate differentiation pathways, while dashed arrows represent signaling pathways and functional roles. Color coding: blue = conventional PKC (cPKC: α, βI, βII, γ); green = novel PKC (nPKC: δ, ϵ, η, θ); orange = atypical PKC (aPKC: ζ, ι/λ).).

In summary, PKC plays a central signaling hub role in hematopoiesis. The balance and activity of different PKC subtypes are crucial for normal hematopoiesis and the pathogenesis of hematological diseases (**Figure 1**).

## 4 Lineage-specific landscapes of PKC signaling

### 4.1 PKC in myeloid leukemias

PKC family plays complex and often contradictory roles in the pathogenesis and treatment response of myeloid leukemia. The specific function of individual PKC isoforms appears to be context-dependent, influencing cell survival, proliferation, drug resistance, and differentiation.

#### 4.1.1 PKC isoforms in acute myeloid leukemia: dual oncogenic/tumor-suppressive functions, drug resistance and therapeutic targeting

PKC is closely related to the occurrence, treatment response, and development of drug resistance in acute myeloid leukemia (AML) (58–61). The activity of PKC may be hijacked by common AML-related lesions. In a significant proportion of patients, the overexpression of the main kinase PDK1 leads to increased phosphorylation and activation of various PKC subtypes (especially PKCα), thereby promoting the survival of leukemia cells and being associated with a poorer clinical prognosis (62). Similarly, the resistance to receptor tyrosine kinase inhibitors (such as those targeting FLT3) is mechanistically related to the compensatory upregulation of AXL and the subsequent enhancement of the PKC signaling pathway, forming a bypass survival pathway that maintains the growth of leukemia cells (59). This makes PKC signaling a convergence node downstream of various genetic and epigenetic alterations, enhancing its oncogenic potential.

Once activated, the PKC family divides into two factions, determining opposite cell fates. On one hand, specific subtypes promote differentiation and apoptosis, acting as potential inhibitors of the malignant phenotype or as mediators of treatment-induced cell death. PKC-δ often functions as a pro-apoptotic effector. It plays a crucial regulatory role in all -trans retinoic acid (ATRA)-induced sensitive cell differentiation responses (59), and through selective pharmacological activation with drugs like ingenol-3-angelate (PEP005), it can effectively induce apoptosis in myeloid leukemia cell lines and primary AML leukemia cells, while protecting normal CD34^+^ precursor cells (63). PKC δ is also involved in cellular drug resistance. Topoisomerase IIβ (TOP2B) is a novel mediator of RA resistance in APL cell lines. The stability and activity of the TOP2B protein are regulated by PKC δ. The combined application of PKC δ inhibitors and RA can disrupt the differentiation arrest of retinoic acid-resistant APL cell lines (64). PKC-α is also involved in promoting monocyte differentiation in certain cases (65), and participates in specific apoptotic pathways, such as the one initiated by tumor necrosis factor-α (TNF-α) (66). On the other hand, an aspect dominated by PKC-ϵ and PKC-ζ functions as a powerful guardian of cell survival and a powerful engine of treatment resistance. PKC-ϵ regulates mitochondrial redox homeostasis to maintain the survival of leukemia cells, otherwise it would trigger excessive reactive oxygen species (ROS) activity leading to cell death (60). Additionally, its overexpression is clinically associated with poor prognosis in patients, and in mechanism, it promotes resistance to chemotherapy by upregulating the expression of drug efflux pump protein P-glycoprotein (ABCB1), thereby reducing the intracellular concentration of drugs like daunorubicin (67). The atypical PKC-ζ isoform counteracts extrinsic cell apoptosis by interfering with the formation of the death-inducing signaling complex (DISC) at the Fas receptor site, effectively weakening death receptor signal transduction (68).

Current therapeutic strategies targeting PKC can be broadly classified into three aspects: selectively activate the apoptotic and differentiating subtypes, inhibition of pro-survival or upstream nodes, and utilization of PKC activity as a predictive biomarker. Agonist strategies such as PEP005 directly activate PKC-δ to trigger cell apoptosis, showing efficacy even in primary acute myeloid leukemia (AML) samples (63). AS101, together with the PKC activators epidermal inhibitory protein-1 and phorbol myristate acetate (PMA), exhibits synergistic differentiation effects on human myeloid leukemia cells *in vitro* and in a mouse model. This differentiation-promoting effect is associated with the actions of PKCα and PKCδ (69). The treatment of arsantin can also promote the differentiation of HL60 cells. This treatment causes the levels of PKC a, PKC bII, and total PKC to increase at the early time points (70). The PKC activator prostratin also has a similar effect, inducing AML cell differentiation and G1 phase cycle arrest by regulating the kinase (ERK) kinase (MEK) through PKC signaling (71). Inhibition strategies are more diverse, such as the broad-spectrum PKC inhibitor enzastaurin, which shows pro-apoptotic activity in AML-derived cell lines and primary cells, possibly through mechanisms such as inhibition of PKC-α and downstream effects on BCL-2 and ERK signaling pathways (72). GSK-J4 was applied to human acute myeloid leukemia (AML) KG-1a cells. By reducing the expression of CyclinD1 and CyclinA2 and increasing the expression of P21, it decreased the cell viability and caused the cell cycle to stall at the S phase. Additionally, GSK-J4 enhanced the expression of proteins related to cell apoptosis (cle-caspase-9 and bax), and inhibited the PKC-a/p-Bcl2 pathway, thereby promoting cell apoptosis (73). At a more upstream level, the multi-kinase inhibitor PKC412 (midostaurin) directly targets driver mutations like FLT3-ITD and inhibits PKC activity, representing a successful dual-target strategy that has now been incorporated into standard treatment regimens (74). Targeted therapy based on the metabolic dependence of drug-resistant cell populations (leukemia stem cells, LSCs) has also been developed recently. Studies have found that the ALDH2-PKCδ-SHMT2 axis can maintain mitochondrial homeostasis in LSCs and promote their self-renewal and chemotherapeutic resistance, In this study based on 40 newly diagnosed, relapsed or remitted AML patients and 20 healthy hematopoietic stem cell samples from donors, PKC δ was confirmed to be a positive signal for stem cell survival, further demonstrating that the PKC complex is environment and background-dependent (61). By using drugs like rafifenalin to disrupt this specific metabolic adaptation and inhibit the interaction between ALDH2-PKCδ, this provides a promising strategy for eliminating the root cause of disease recurrence. In addition to direct targeting, the activation state of PKC can also provide information for treatment options. For example, the novel phospholipid mimetic prodrug KPC34 is only activated by phospholipase C, and the activity of this enzyme is coupled with PKC signal transduction. Therefore, AML cells with activated PKC are more sensitive to KPC34, making PKC activation a predictive marker for the efficacy of this drug (75).

The PKC signaling in AML is complex and not absolute but changes with the environment. Even specific PKC subtypes may exhibit different functions in different cell contexts; for example, the activity of PKC-α is related to differentiation induction and Bcl-2 phosphorylation associated with poor survival rates (65, 76). In the future, acute myeloid leukemia (AML) treatment may attempt to analyze the activation state of PKC isoforms in patients’ bodies in advance, target the stimulation of “therapeutic” PKC pathways, and simultaneously inhibit “pathogenic” pathways. Combined with conventional chemotherapy, such as differentiation agents like ATRA (65, 77), or other targeted therapies, it ultimately overcomes drug resistance and improves the long-term prognosis of patients.

#### 4.1.2 PKC isoforms in chronic myeloid leukemia: BCR-ABL-independent TKI resistance, dual functions and therapeutic strategies

PKC is deeply involved in the occurrence and development of chronic myeloid leukemia (CML), especially in the formation of treatment resistance. In CML, one of the main and clearly defined functions of PKC is its significant contribution to the resistance to BCR-ABL tyrosine kinase inhibitors (TKIs), which represents a major clinical challenge. Among the subtypes of PKC, PKC-β has been identified as the main regulatory factor for TKI resistance. Its overexpression is closely related to imatinib resistance, and inhibiting the PKC-β gene or using drug means can restore the sensitivity of TKI-resistant CML cell lines and primary CD34^+^ cells (78). The mechanism is summarized as the PKC-β/Alox5/PTEN survival promotion axis: activated PKC-β signals through the ERK1/2 pathway, upregulates 5-lipoxygenase (Alox5), the upregulated Alox5 leads to the inactivation of tumor suppressor PTEN, bypassing the action of BCR-ABL, and maintaining cell proliferation and survival in the presence of tyrosine kinase inhibitors (TKIs) (78). *In vivo*, the selective PKC-β inhibitor LY333531 significantly prolonged survival in a patient-derived xenograft (PDX) model of CML, providing a promising strategy to overcome this form of resistance (78).

In addition to PKC-β, other PKC subtypes also promote TKI resistance through different and complementary mechanisms. For example, low-dose use of the broad-spectrum PKC inhibitor staurosporine has been shown to selectively reverse imatinib resistance independent of BCR-ABL. This sensitization effect is mainly achieved by inhibiting PKC-α rather than PKC-β. Inhibiting PKC-α induces significant G2/M phase cell cycle arrest by downregulating key mitotic regulators (such as CDC23), which makes the originally resistant chronic myeloid leukemia cells sensitive to imatinib-induced apoptosis, revealing the crucial role of PKCα in regulating cell cycle checkpoints mediated by survival in the context of therapeutic stress (79). There is also a connection between the oncogene-driven PKC signal and BCR-ABL. This can be exploited in treatment. Natural compounds such as phenethyl isothiocyanate (PEITC) demonstrate the potential for dual targeting, namely simultaneously inhibiting BCR-ABL and several PKC subtypes (including α, βII, and ζ). This synergistic inhibition disrupts the cross-talk between these pathways and their common downstream effectors such as Raf1 and ERK1/2, thereby significantly enhancing the cytotoxic effect of imatinib (80).

The influence of PKC in chronic myeloid leukemia is not only reflected in the development of drug resistance; it is also a key regulator of fundamental cellular processes, such as differentiation and programmed cell death. The activation of PKC is associated with megakaryocytic differentiation in chronic myeloid leukemia (CML)-derived K562 cells induced by phosphatidylcholine esterase (TPA). This differentiation process requires the novel PKC (nPKC)-MAPK pathway, which drives cell differentiation by downregulating nuclear lamina protein B23 (a nucleolar protein) through proteasome-mediated nuclear lamina protein B23 (a nucleolar protein) downregulation, which is closely related to cell differentiation determination (81). The overexpression of PKCι leads to an increase in resistance to drug-induced apoptosis, while inhibiting the expression of PKCι makes cells more sensitive to drug-induced apoptosis (82). Conversely, the activation of PKC can also serve as a trigger for eliminating leukemia cells. The nucleoside analogue acalycitin induces a novel form of autophagic cell death in CML cells, a process that strictly depends on the activation of PKC. This effect can be completely eliminated by classical and novel PKC inhibitors (GF109203X and Ro-32-0432) (83). This PKC-dependent autophagic death pathway provides a valuable alternative mechanism for targeting CML cells (including those with TKI resistance). Perhaps more importantly, PKC plays a role in targeting the resistant leukemia stem cell (LSC) subpopulation. The farnesyltransferase inhibitor BMS-214662 selectively induces mitochondrial apoptosis in the original CD34^+^38^-^ CML stem/progenitor cells. This powerful effect is triggered by the upregulation of specific PKCβ, which precedes Bax conformational changes, mitochondrial dysfunction, and caspase activation. The crucial role of PKCβ has been confirmed, as protein kinase regulator agents like broxistatin-1 can block this apoptotic cascade, thereby establishing that the activation of PKCβ is a key step in eliminating LSCs and is a modifiable step that can be intervened by drugs (84).

**Figure 2:**
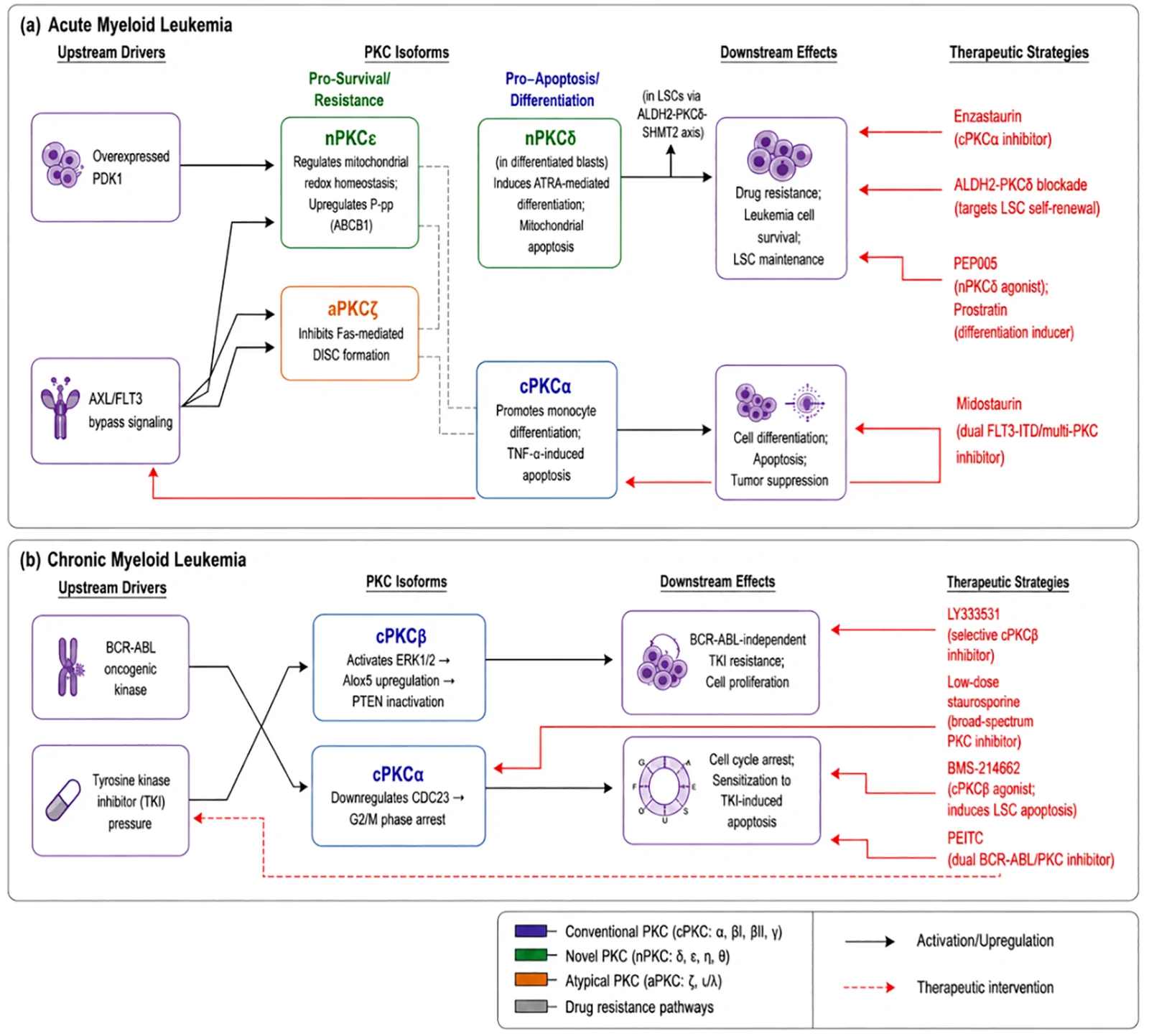
Lineage-specific PKC signaling in myeloid leukemias. This schematic illustrates the context-dependent dual roles of protein kinase C (PKC) isoforms in myeloid leukemias, structured as upstream oncogenic drivers, PKC-mediated signaling cascades, downstream biological effects, and corresponding therapeutic strategies. In acute myeloid leukemia (AML, panel **(a)**), PKC isoforms are functionally segregated into two opposing groups: pro-survival/resistance isoforms (nPKCϵ, aPKCζ) that mediate drug efflux, mitochondrial redox homeostasis, and leukemia stem cell (LSC) maintenance, and pro-apoptosis/differentiation isoforms (nPKCδ, cPKCα) that induce all-trans retinoic acid (ATRA)-mediated differentiation and mitochondrial apoptosis. Notably, nPKCδ exhibits a lineage-specific dual function: it promotes apoptosis in differentiated leukemic blasts but supports LSC self-renewal via the ALDH2-PKCδ-SHMT2 axis. In chronic myeloid leukemia (CML, panel **(b)**), PKC signaling drives BCR-ABL-independent tyrosine kinase inhibitor (TKI) resistance. cPKCβ mediates resistance through the ERK1/2-Alox5-PTEN axis, while cPKCα induces G2/M phase arrest and sensitizes cells to TKI-induced apoptosis. Therapeutic strategies include selective isoform inhibition, pro-apoptotic PKC activation, dual oncogene/PKC targeting, and LSC-specific elimination via PKCβ agonism. Solid black arrows indicate activation/upregulation, and red dashed arrows represent therapeutic interventions. Color coding: blue = conventional PKC (cPKC); green = novel PKC (nPKC); orange = atypical PKC (aPKC).

PKCβ and PKCα are key components of BCR-ABL-independent TKI resistance. At the same time, the PKC pathway has the contradictory ability to be used for therapeutic benefits, mediating differentiation programs and alternative cell death pathways such as autophagy. It is well known that the interaction between BCR-ABL and PKC further consolidates their interdependent relationship in maintaining the leukemia state. Targeting specific PKC subtypes - whether through selective inhibition of the subtype driving resistance or through drug-mediated activation of PKC to induce lethal differentiation or death under specific circumstances - is a highly promising adjunctive treatment approach. Future treatment strategies for chronic myeloid leukemia, especially to overcome resistance and prevent recurrence, are likely to involve the rational combination of tyrosine kinase inhibitors with PKC modulators of different subtypes, which will be personalized based on the dominant resistance mechanism active in individual patients (**Figure 2**).

### 4.2 Function of PKC subtypes in acute lymphoblastic leukemia

Acute lymphoblastic leukemia (ALL) is classified into T-cell acute lymphoblastic leukemia (T-ALL) and B-cell acute lymphoblastic leukemia (B-ALL) (85). A large number of studies have shown that different isoforms of the PKC family do play distinct and even opposite roles in B-ALL and T-ALL.

Aberrant activation of Notch3 signaling is one of the key pathogenic mechanisms of T-ALL (86). PKCθ is a core downstream effector molecule of the Notch3 signaling pathway (87–89). In the Notch3-IC transgenic mouse model, the activity of PKCθ is significantly enhanced, and its membrane translocation and activation are dependent on the presence of a functional pre-T cell receptor (pre-TCR). Mechanistically, Notch3 upregulates pre-TCR expression, recruits the Src family kinase Lck to bind to PKCθ, and promotes the phosphorylation and activation of PKCθ; the activated PKCθ further initiates the NF-κB signaling pathway by phosphorylating the IKK complex, thereby maintaining the malignant proliferation of T-ALL cells. Knockout of PKCθ can significantly reduce the incidence of T-ALL induced by Notch3 and decrease the expression of NF-κB target genes (such as Bcl-2 and c-Myc), confirming that PKCθ is an essential molecule for Notch3-mediated T-ALL development (90).

Chalcone derivatives have been previously shown to inhibit Notch signaling in various T-ALL cells and induce cell cycle arrest and apoptosis (91, 92). Moreover, chalcone derivatives significantly alter the protein expression level of Rack1 after acting on T-ALL cells. Overexpression of Rack1 enhances PKCα activity, thereby leading to T-ALL cell resistance to classic chemotherapeutic drugs such as vincristine and prednisone. This is because Rack1 directly binds to PKCα, promotes its translocation to the cell membrane and maintains its active conformation, thereby downregulating the expression of apoptosis-related molecules FEM1b, Apaf-1, and caspase 3, and inhibiting chemotherapy-induced cell apoptosis (93). The role of PKCα in T-ALL presents a seemingly contradictory phenomenon that may actually reflect the complexity of its functions: in cell lines, increased activity of PKCα promotes chemotherapy resistance (94); while in patients, decreased overall expression level of PKCα is associated with extremely poor clinical prognosis. Among pediatric T-ALL patients already classified as high-risk (MRD-HR), low PKCα expression is an independent poor prognostic factor, which is correlated with ultra-high recurrence rate and low survival rate (95).

Chalcone derivatives can inhibit the membrane translocation and activity of PKCβ, thereby downregulating its downstream Akt/GSK3β/β-catenin pro-survival signaling pathway and inducing cell apoptosis (96). Notch1 signaling negatively regulates the expression of PKCβ, and inhibition of PKCβ can restore the sensitivity of drug-resistant cells to drugs. Aberrant activation of PKCβ is one of the mechanisms by which T-ALL cells develop resistance to dexamethasone, which provides clues for clinical chemotherapy failure (97). Therefore, the role of PKCβ in T-ALL (pro-apoptotic or pro-survival) may depend on its upstream signaling status and the cellular microenvironment.

The core role of PKCδ in T-ALL is to act as a key “drug resistance regulatory node”, particularly involved in mediating resistance to Notch1-targeted therapy (94). When Notch signaling is inhibited, it is activated from a “latent” state to a pro-survival drug resistance hub, which is different from the action modes of PKCθ (directly driving proliferation) and PKCα (mediating chemotherapy resistance) (94). In T-ALL, high expression of PKCζ is thought to stabilize the mismatch repair protein MSH2, which may help enhance the therapeutic response to thiopurine drugs (such as 6-MP/6-TG) (98). This also suggests that the expression level of PKCζ may serve as a potential biomarker for predicting chemotherapy efficacy. The specific role and clinical significance of PKCι/λ (PKCι in humans and PKCλ in mice) in T-ALL remain unknown.

Philadelphia chromosome-positive (Ph^+^) leukemia is mainly divided into two major subtypes: Ph^+^ acute lymphoblastic leukemia (Ph^+^ ALL) and blast crisis of chronic myeloid leukemia, among which Ph^+^ acute lymphoblastic leukemia is mainly B-cell predominant (98). In sharp contrast to the pro-resistance role of PKCδ in T-ALL, its core function in B-ALL is to act as a key “safety valve” or negative feedback regulator, aiming to limit the excessive activation of oncogenic signaling pathways. Ph^+^ B-ALL progresses due to the continuous activation of oncogenic tyrosine kinases (such as BCR-ABL1). Studies have found that PKCδ is selectively activated by tyrosine kinases in this subtype, and recruits phosphatases such as SHP1 and SHIP1 to form a negative feedback loop by phosphorylating the S268 and T271 sites of CD25, thereby maintaining kinase signal homeostasis. As the α chain of the IL-2 receptor, phosphorylation of CD25 can enhance its binding capacity to phosphatases, inhibit excessively activated tyrosine kinase signaling, and prevent cells from undergoing apoptosis due to signal imbalance. Preclinical studies have shown that antibody-drug conjugates (ADCs) targeting CD25 can target the PKCδ-CD25 pathway and induce complete remission in patient-derived xenograft (PDX) models of Ph^+^ B-ALL, providing a new therapeutic direction for drug-resistant patients (99, 100). Furthermore, another study has shown that the activation of PKCδ in Ph+ B ALL can induce cell cycle arrest and differentiation (101). High levels of PKCϵ transcripts have been detected in both cells and cell lines from Ph^+^ ALL patients. Aberrantly high expression of PKCϵ is associated with aggressive Ph^+^ leukemia types, exerts a protective effect on apoptosis induced by BCR-ABL inhibition (such as imatinib treatment), and is a mechanism leading to treatment failure and drug resistance (102).

Activation of PKC (including PKCα/β) in bone marrow stromal cells can protect B-ALL cells from chemotherapeutic drug killing by inhibiting the activity of ABC transporters on leukemia cells (103). This study indicates that PKC signaling in stromal cells of the tumor microenvironment (rather than leukemia cells themselves) is a key factor in the development of drug resistance in B-ALL. In the Ph^+^ B-ALL model, the activity of BCR-ABL kinase inhibits the catalytic activity of PKCα. This inhibitory effect helps enhance intracellular calcium signaling, thereby potentially promoting the proliferation and survival of leukemia cells and inhibiting apoptosis (104). Different from the definite pro-chemoresistance role of PKCα in T-ALL, its role in B-ALL is context-dependent. In the early stage of B-cell development, it may act as a “guardian” (tumor suppressor); in the tumor microenvironment, PKCα in stromal cells is an “accomplice” (promoting drug resistance); while in Ph^+^ B-ALL cells, it may be “hijacked” by oncogenic kinases to become part of the survival signal.

PKCβ is a key kinase for pre-BCR signal transduction. Inhibition of PKCβ rapidly reduces the phosphorylation of AKT and GSK3β, leading to abnormal accumulation of β-catenin, thereby downregulating c-Myc and upregulating c-Jun, and ultimately inhibiting cell growth. Among them, pro-B ALL cell lines carrying t (4;11) translocation are the most sensitive to PKCβ inhibition (105, 106). Stromal cell PKCβ controls the expression of adhesion/stromal proteins, thereby activating the PI3K and ERK pathways in tumor cells, stabilizing the anti-apoptotic protein BCL-XL signal, and activating the PKCβ-dependent transcription factor EB to regulate lysosomal biosynthesis and membrane integrity, thereby significantly reducing the efficacy of chemotherapeutic drugs (107).

**Figure 3:**
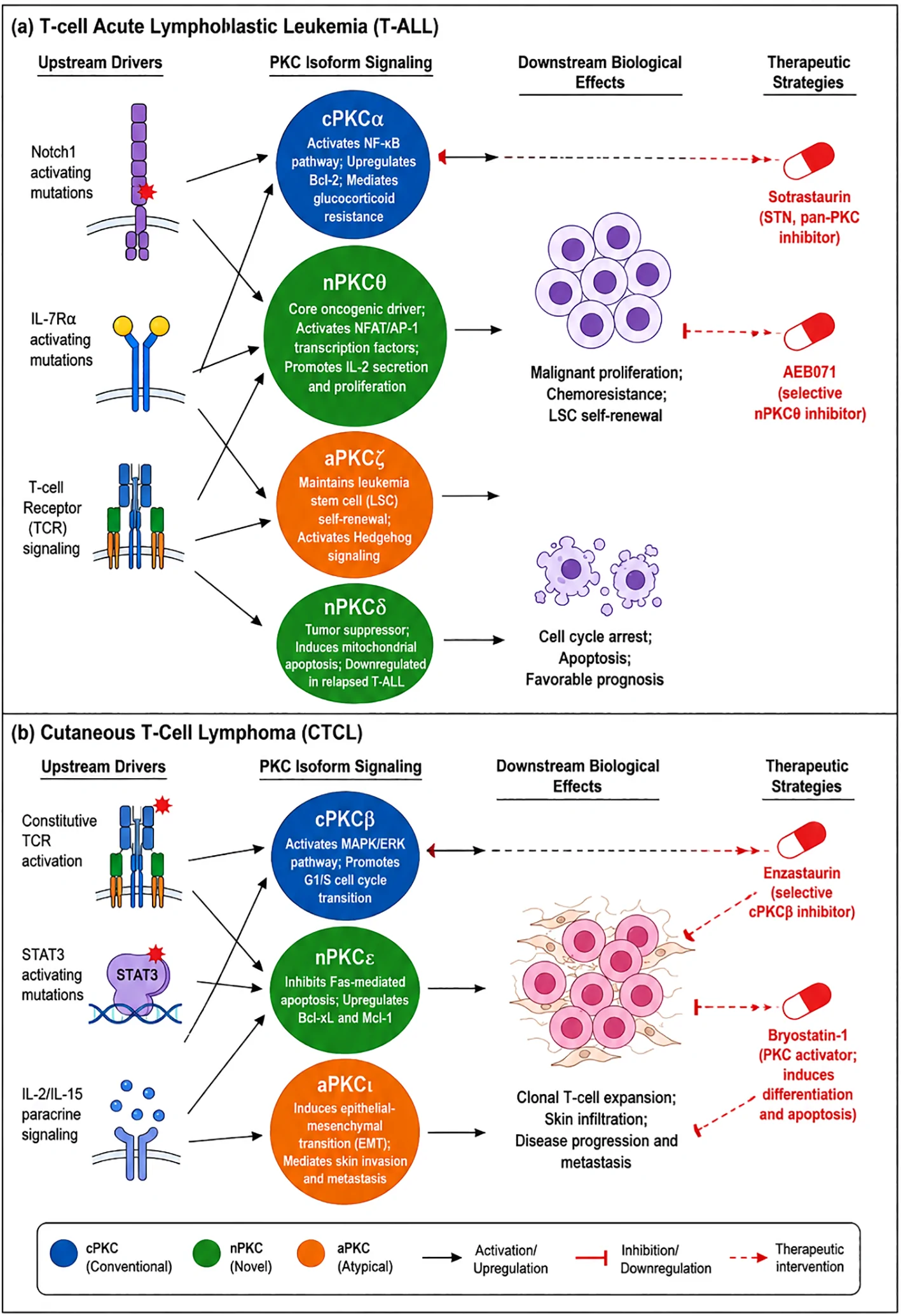
Protein kinase C (PKC) signaling pathways in t-cell malignancies. Protein kinase C isoforms exert context-dependent oncogenic or tumor-suppressive functions in T-cell malignancies, representing promising therapeutic targets. **(a)** In T-cell acute lymphoblastic leukemia (T-ALL), activating mutations in Notch1, IL-7Rα and constitutive T-cell receptor (TCR) signaling drive aberrant activation of multiple PKC isoforms. Conventional PKCα (cPKCα) mediates glucocorticoid resistance via NF-κB/Bcl-2 pathway activation. Novel PKCθ (nPKCθ) acts as a core oncogenic driver by activating NFAT/AP-1 transcription factors and promoting IL-2-dependent proliferation. Atypical PKCζ (aPKCζ) maintains leukemia stem cell (LSC) self-renewal through Hedgehog signaling. In contrast, nPKCδ functions as a tumor suppressor by inducing mitochondrial apoptosis and is frequently downregulated in relapsed T-ALL. Therapeutic strategies include the pan-PKC inhibitor sotrastaurin (targeting cPKCα and nPKCθ) and the selective nPKCθ inhibitor AEB071. **(b)** In cutaneous T-cell lymphoma (CTCL), constitutive TCR activation, STAT3 mutations and IL-2/IL-15 paracrine signaling promote malignant transformation. cPKCβ drives cell cycle progression via MAPK/ERK pathway activation. nPKCϵ inhibits Fas-mediated apoptosis by upregulating anti-apoptotic proteins Bcl-xL and Mcl-1. aPKCι mediates skin invasion and metastasis by inducing epithelial-mesenchymal transition (EMT). Therapeutic interventions include the selective cPKCβ inhibitor enzastaurin and the pan-PKC activator bryostatin-1, which induces differentiation and apoptosis of malignant T cells.

PKCθ is highly expressed in specific genetic subtypes of B-ALL (such as TCF3 rearrangement), and this high expression is associated with increased sensitivity to thiopurine drugs (such as 6-MP/6-TG). However, its high expression is a correlative marker rather than a direct causal factor. Knockdown of PKCθ reduces MSH2 protein but does not directly cause drug resistance, indicating that PKCθ serves as a correlative marker for drug sensitivity to thiopurines (98). The role of PKCζ in B-ALL is mainly reflected in regulating the interaction between leukemia cells and the bone marrow microenvironment, and affecting chemotherapy sensitivity in specific genetic subtypes, but its mechanism of action is often correlative rather than directly causally driven (98). As a key downstream kinase of the SDF-1/CXCR4 signaling pathway, PKCζ regulates the chemotaxis, adhesion, and homing of leukemia precursor B cells to the bone marrow stroma (108). In pediatric B-ALL with TCF3 rearrangement, the mRNA and protein levels of PKCζ and its truncated active form PKMζ are significantly upregulated (98) (**Figure 3**).

### 4.3 Function of PKC subtypes in chronic lymphocytic leukemia

Chronic lymphocytic leukemia (CLL) is a mature B-cell malignancy characterized by the accumulation of lymphocytes in the peripheral blood, bone marrow, and lymphoid tissues. PKCβII and PKCδ are the most extensively studied isoforms in this disease, with significantly elevated mRNA and protein levels. Introduction of a kinase-inactive PKCα mutant (PKCα-KR) into hematopoietic stem/progenitor cells leads to the development of CLL-like disease in mice. Impaired PKCα function results in a significant upregulation of its “counterpart” PKCβII, and this upregulation is essential for CLL development; knockdown of PKCβ can block leukemic transformation (109–111). PKCβII is highly expressed in CLL cells, and its expression level is positively correlated with disease aggressiveness. The selective PKCβ inhibitor MS-553, by inhibiting BCR and Wnt/β-catenin signaling pathways and overcoming stromal cell protection, can delay disease progression and prolong survival in the mouse CLL model (112). In ZAP70-positive CLL patients, PKCβII is persistently localized to the membrane lipid raft region, inhibiting cell apoptosis by phosphorylating the anti-apoptotic protein BCL2 and promoting the ubiquitin-dependent degradation of the pro-apoptotic protein BIMEL. In addition, PKCβII can upregulate the secretion of cytokines such as IL-6 and IL-10 by activating the NF-κB pathway, forming a pro-survival positive feedback loop between the tumor microenvironment and tumor cells. Preclinical studies have confirmed that Enzastaurin, a selective PKCβ inhibitor, can reduce the phosphorylation level of BCL2, restore the pro-apoptotic function of BIMEL, significantly inhibit tumor growth in CLL animal models, and enhance the efficacy of chemotherapeutic drugs such as fludarabine (113).

In contrast to the oncogenic function of PKCβII, PKCδ mainly exerts a pro-apoptotic effect in CLL. Western blot analysis showed that the protein level of PKCδ is significantly elevated in CLL patient cells, and its activity is positively correlated with patient prognosis. Mechanistically, PKCδ can directly phosphorylate the Ser15 site of p53, enhance the transcriptional activity of p53, upregulate the expression of downstream target genes such as p21 and PUMA, and induce cell cycle arrest and apoptosis. Treatment with the PKCδ inhibitor rottlerin or knockdown of PKCδ by siRNA can significantly inhibit CLL cell apoptosis, while overexpression of PKCδ enhances cell sensitivity to chemotherapeutic drugs. In addition, PKCδ can also affect CLL cell survival by regulating the autophagic pathway, but its specific role in autophagy (promotion or inhibition) remains controversial and needs further investigation (109, 110, 112).

The expression of PKCϵ is significantly upregulated in CLL cells, and it is abnormally localized to the nucleus in large quantities driven by Lyn kinase (a key kinase in the BCR signaling pathway). Nuclear PKCϵ can transcriptionally activate multiple key anti-apoptotic genes (such as Mcl-1, XIAP, and Bcl-2), and promote NF-κB activation and VEGF production, thereby leading to apoptotic defects and chemotherapy resistance in CLL cells (109, 111, 113). Compared with normal B cells, the expression of PKCζ is upregulated in CLL cells. Inhibition of overall PKC activity, including PKCζ, rapidly induces CLL cell apoptosis and downregulates key survival proteins such as CREB and Daxx, suggesting that PKCζ is an important node in the signal network on which CLL cells depend for survival (115).

### 4.3 Function of PKC subtypes in diffuse large B-cell lymphoma

Diffuse large B-cell lymphoma (DLBCL) is the most common subtype of non-Hodgkin lymphoma. According to the gene expression profile, it is divided into germinal center B-cell-like (GCB) and activated B-cell-like (ABC) subtypes. PKCβ and PKCϵ exhibit subtype-specific functions in this disease. ABC-DLBCL undergoes malignant proliferation due to persistent activation of the NF-κB pathway. Studies have found that CD79A/B mutations are the main driving factors for NF-κB activation in this subtype, and PKCβ is a key molecule for signal transduction mediated by CD79 mutations. Mechanistically, CD79 mutations lead to abnormal activation of B-cell receptor (BCR) signaling, generate diacylglycerol (DAG) through the BTK-PLCγ pathway, and recruit PKCβ to the cell membrane; the activated PKCβ further phosphorylates CARD11, promotes the assembly of the CBM (CARD11-BCL10-MALT1) complex, and ultimately initiates the NF-κB pathway. Sotrastaurin (STN), a selective PKC inhibitor, can target and inhibit PKCβ activity, induce G1 phase cell cycle arrest and apoptosis in CD79-mutated ABC-DLBCL cell lines (such as TMD8 and HBL1), but is ineffective in CARD11-mutated DLBCL, confirming that the role of PKCβ depends on CD79-mediated upstream signaling (116, 117).

**Figure 4:**
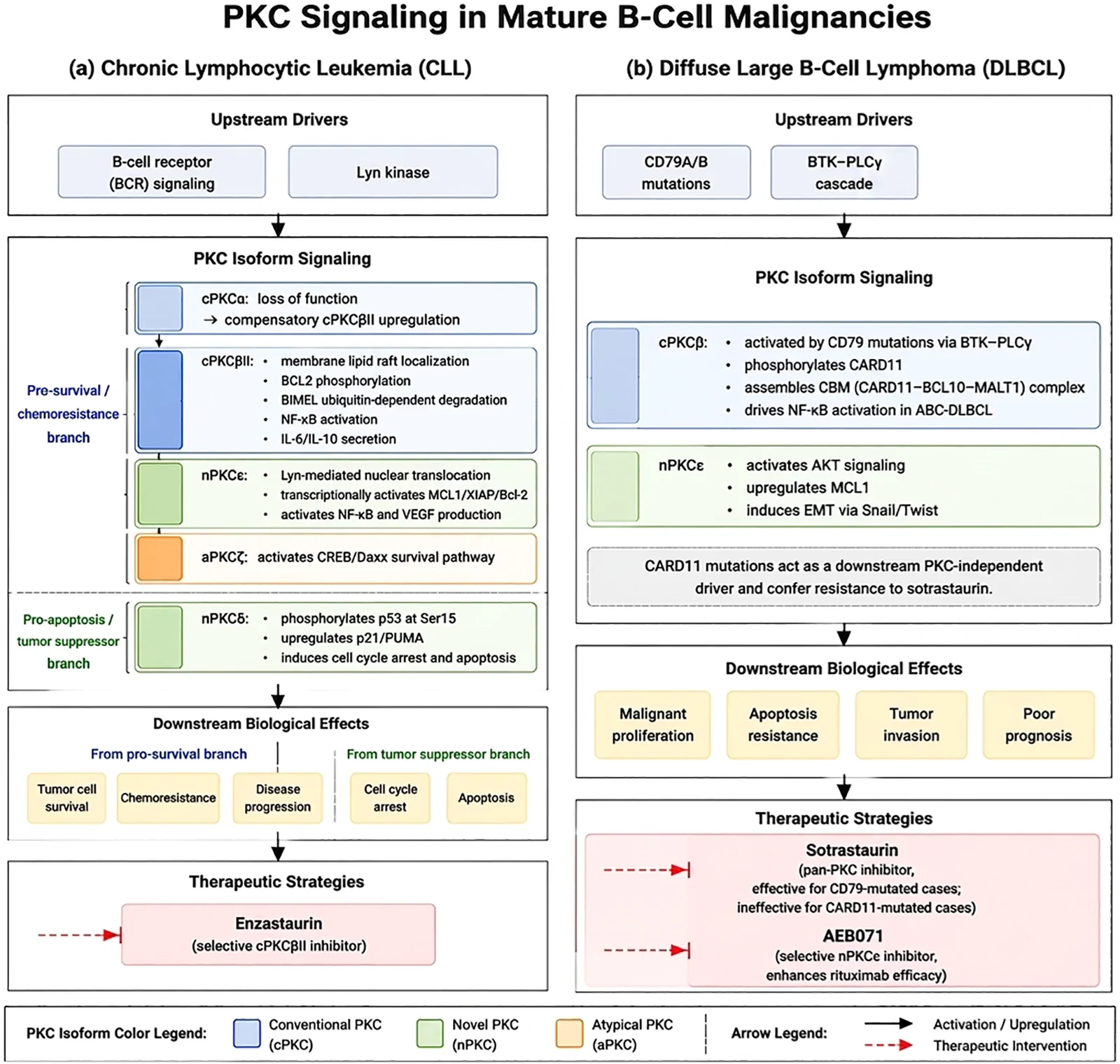
PKC signaling in mature B-cell malignancies. This schematic illustrates the isoform-specific and context-dependent functions of protein kinase C (PKC) signaling in mature B-cell malignancies, structured as upstream oncogenic drivers, PKC-mediated signaling cascades, downstream biological effects, and corresponding therapeutic strategies. In chronic lymphocytic leukemia (CLL, panel a), PKC isoforms are functionally segregated into two opposing branches. Loss of conventional PKCα (cPKCα) function triggers compensatory upregulation of cPKCβII; cPKCβII, novel PKCϵ (nPKCϵ) and atypical PKCζ (aPKCζ) collectively drive tumor cell survival, chemoresistance and disease progression via BCL2 phosphorylation, BIMEL ubiquitin-dependent degradation, NF-κB activation and CREB/Daxx signaling. In contrast, nPKCδ acts as a tumor suppressor by phosphorylating p53 at Ser15, upregulating p21/PUMA, and inducing cell cycle arrest and apoptosis. The selective cPKCβII inhibitor enzastaurin is a promising targeted agent for CLL. In diffuse large B-cell lymphoma (DLBCL, panel b), CD79A/B mutations drive aberrant cPKCβ activation through the BTK-PLCγ cascade, which phosphorylates CARD11, assembles the CBM (CARD11-BCL10-MALT1) complex, and initiates NF-κB signaling in the ABC subtype. nPKCϵ promotes apoptosis resistance and tumor invasion via the AKT-MCL1 axis and epithelial-mesenchymal transition (EMT). Notably, CARD11 mutations act as a PKC-independent downstream driver and confer resistance to the pan-PKC inhibitor sotrastaurin. The selective nPKCϵ inhibitor AEB071 serves as an adjuvant agent that enhances rituximab efficacy. Solid black arrows indicate activation/upregulation, and red dashed arrows represent therapeutic interventions. Color coding: blue = conventional PKC (cPKC); green = novel PKC (nPKC); orange = atypical PKC (aPKC).

PKCϵ is highly expressed in some DLBCL cell lines and clinical samples, and its expression level is associated with poor prognosis of patients. Mechanistically, PKCϵ upregulates the expression of the anti-apoptotic protein MCL1 by activating the AKT signaling pathway, thereby inhibiting cell apoptosis. *In vitro* experiments have shown that knockdown or inhibition of PKCϵ using PKCϵ-specific siRNA or inhibitors (such as AEB071) can significantly reduce the proliferative activity of DLBCL cells and enhance the anti-tumor effect of rituximab. In addition, PKCϵ can also enhance the invasiveness of DLBCL cells by regulating the expression of epithelial-mesenchymal transition (EMT)-related molecules (such as Snail and Twist), providing a new perspective for the study of metastasis mechanisms in this subtype (118) (**Figure 4**, **Table 1**).

**Table 1:** The role of PKC isoforms in different types of leukemia.

PKC isoforms	Mechanism	Diseases	Research materials	Reference
PKC α	Cell survival (Overexpression of PDK1 activates PKCα)	AML	Mononuclear cells from AML patients	(62)
	Differentiation	AML	THP-1	(65)
		APL, AML	HL-60, U937 and THP-1	(69)
		APL	HL-60	(70)
	Apoptosis	AML; CML; APL	OCI-AML2 and OCI-AML3; U937, KG1, K562, THP-1, and HL60	(72)
		AML	KG-1a	(73)
	Drug resistance	AML	AML patients sample, OCI-AML3	(76)
	Cell cycle arrest at the G2/M phase	CML	Human CML parent cell line K562 and IM-resistant cell line K562R	(79)
	Drug resistance	T-ALL	DND41, KOPTK1, TALL-1	(91–94)
	Low survival	T-ALL	Children (MRD-HR)	(95)
	Loss of function	B-ALL	B-ALL patients samples	(103, 104)
		CLL	CLL patient samples	(111)
PKC β	Drug resistance	CML	LAMA‐84 and KU‐812, K562 and K562R	(78)
	Apoptosis	APL	HL-60, HL525	(66)
		CML	Patient samples (Isolation of CD34^+^ and CD34^+^38^-^ cells)	(84)
	Differentiation	APL	HL-60	(70)
	Tumor growth	T-ALL	CCRF-CEM	(96)
		B-ALL	RS4;11, SEM-K2, HB-1119, REH, TOM-1, SUP-B15, NALM-6	(105, 106)
		CLL	EMC2, EMC4, EMC6	(111)
		DLBCL	SUDHL-4, OCI-LY8	(116, 117)
	Drug resistance	T-ALL	REH	(97)
		B-ALL	NALM-6, BL-41	(107)
		CLL	Patient samples	(112)
PKC δ	Drug resistance	T-ALL	MOLT-3, DND-41, HPB-ALL, ALL-SIL, PEER, JURKAT, DND-41, and ALL-SIL	(94)
		T-ALL	CUTLL1, Jurkat, PDTALL13c	(98)
		APL	NB4-MR2 and NB4	(64)
	Apoptosis	AML	HL60, NB4, U937, K562, and KG1a	(63)
	Differentiation	APL	NB-4, HL-60	(77)
	Regulator	B-ALL	Patient-derived B-ALL and AML samples	(99, 100)
PKC ϵ	Promote survival and development	AML	Patient-derived AML samples	(60)
	Drug resistance	AML	AML patient samples;U937 and HEL	(67)
	Tumor growth, Drug resistance	B-ALL	Clinical samples	(102)
		CLL	EMC2, EMC4, EMC6, clinical samples	(114)
PKCθ	tumor growth	T-ALL	Primary cells of mice	(90)
	Drug resistance	B-ALL	Clinical samples	(98)
PKC ζ	Fas-mediated death signal	APL, AML, T-ALL	KG1a, U937, and Jurkat	(68)
	Biomarkers	T-ALL	ALL patient samples	(98)
	Regulator	B-ALL	G2, U937, human CB CD34^+^ cells	(108)
	Tumor growth	CLL	CLL patient samples	(115)
PKC ι	Apoptosis	CML	K562	(82)
PKC (Unspecified subtype)	Drug resistance	AML	MOLM-13 and MV4-11	(59)
	Apoptosis, drug resistance	AML	MFL2 and MR2; OCI-AML3, HL60, and K562	(75)
	Differentiation	AML	HL-60 and U937	(71)
		CML	K562	(81)
	Autophagic cell death	CML	K562, LAMA-84 and JURL-MK1	(83)
	Enhance the toxicity of the drug	CML	K562, KU812	(80)

## 5 Discussion and conclusion

The serine/threonine kinases within the protein kinase C (PKC) family constitute a pivotal signaling nexus that orchestrates a multitude of cellular processes within the hematopoietic system. These kinases are instrumental in preserving cell lineage-specific homeostasis during normal hematopoietic development and are implicated in the pathogenesis of leukemia. This article provides an in-depth analysis of the multifaceted roles of PKC, encompassing its regulatory functions in normal hematopoietic cell production, its contribution to leukemogenesis, its role in mediating drug resistance, and the potential for developing subtype-specific therapeutic strategies.

During normal hematopoiesis, the various subtypes of PKC exhibit significant lineage specificity and function as precise regulators of cell fate. For instance, PKCϵ and PKCλ/ι are crucial for granulocyte differentiation, while PKCα plays a role in M-CSF-induced monocyte differentiation. In erythropoiesis, PKCϵ contributes to the survival of progenitor cells; and in megakaryocytic generation, the transient activation of PKCϵ is crucial for the early determination of the cell lineage. This specificity ensures a coordinated response to cytokine signals, promoting the correct development of each blood cell lineage.

In leukemia, the precisely regulated cellular network becomes disrupted, resulting in functional contradictions. A central theme is the environment-dependent duality observed in individual subtypes. For instance, in acute myeloid leukemia (AML), protein kinase C delta (PKCδ) may function as a tumor suppressor, as its pharmacological activation triggers apoptosis. In contrast, within leukemia stem cells (LSCs), PKCδ interacts with aldehyde dehydrogenase 2 (ALDH2) to preserve mitochondrial homeostasis, thereby facilitating self-renewal and conferring resistance to chemotherapy. This suggests that the same subtype can dictate opposing cellular outcomes—apoptosis or survival—based on the cellular context, which includes factors such as the stage of differentiation and the presence of coexisting mutations.

Furthermore, the balance between antagonistic subtypes is often disrupted in leukemia. In AML, there is typically a balance between pro-apoptotic subtypes (such as PKCδ) and pro-survival subtypes (such as PKCϵ). Cancer stress typically promotes this balance towards survival; for example, excessive expression of PKCϵ enhances chemotherapy resistance by upregulating drug efflux pumps and maintaining redox balance. Similarly, in chronic myeloid leukemia (CML), PKCβ activation forms a powerful survival axis, bypassing BCR-ABL inhibition; while in chronic lymphocytic leukemia (CLL), PKCβ’s physiological role in BCR signaling is overactivated to maintain tumor cell survival. This illustrates that the normal physiological function of PKC can be modified to serve pathological purposes. In lymphocytic leukemia, the role of PKC family members is extremely complex. Different subtypes can exert various effects ranging from “tumor suppression” to “treatment resistance” in T-cell and B-cell leukemias. The function of PKC subtypes is highly dependent on the specific environment. For example, PKCδ acts as a “safety valve” to maintain the survival of Ph^+^ B-ALL cancer cells (99), but under the action of PMA, it can transform into a tumor suppressor (101). PKCα promotes resistance in T-ALL but may play a “guardian” role in the early stage of B-cell development (99). Treatment strategies vary greatly depending on different subtypes and disease backgrounds.

The clinical application of first-generation pan-PKC inhibitors such as midostaurin (PKC412) provides important lessons. Although mitoxantrone is effective in FLT3-mutated acute myeloid leukemia (AML) due to its ability to simultaneously inhibit FLT3 and PKC, its broad-spectrum activity is a double-edged sword. Simultaneously inhibiting the antagonistic isotypes of PKC is likely to lead to a counteracting effect and increase toxicity.

Future research directions in the targeting of protein kinase C (PKC) in leukemia should focus on advancing beyond non-specific inhibition towards sophisticated, precisely-guided strategies. This involves the development of drugs capable of selectively targeting specific PKC isoforms according to leukemia subtypes. In the context of myeloid malignancies, particularly acute myeloid leukemia (AML), the development of specific inhibitors for PKCϵ may prove beneficial in reversing multidrug resistance. Conversely, specific agonists for PKCδ could be employed to induce apoptosis under certain conditions. Regarding lymphoid malignancies, such as chronic lymphocytic leukemia (CLL) and B-cell acute lymphoblastic leukemia (B-ALL), the use of PKCβ inhibitors, like enzastaurin, remains a viable strategy to counteract the hyperactive B-cell receptor (BCR) signaling, potentially in combination with Bruton’s tyrosine kinase (BTK) inhibitors. Additionally, targeting leukemia stem cells (LSCs) is essential for preventing disease recurrence. A promising approach involves targeting specific dependencies of LSCs, such as the ALDH2-PKCδ axis in AML, to effectively eliminate the root cause of the disease.

The PKC signaling network crucially influences cell fate in hematopoiesis, ensuring proper differentiation and function in healthy cells. In leukemia, it aids proliferation, drug resistance, and stem cell maintenance. Advances from broad-spectrum inhibitors to precise therapies highlight our growing understanding. Future leukemia treatments will likely involve tailored PKC regulators to disrupt disease while preserving normal function, aiming to convert PKC from a disease driver to a therapeutic target.
